# Food allergy can be a major hidden cause of chronic asthma which can be uncovered or excluded by introducing a few foods diet

**DOI:** 10.1186/2045-7022-3-S1-O10

**Published:** 2013-05-03

**Authors:** Harry Morrow Brown

**Affiliations:** 1 Independent allergist, UK

## 

Case-histories of three severe chronic asthmatics are presented to draw attention to the possibility that even when there seems to be convincing evidence from history, skin tests, and immunology that dust mite is the major causative allergen, it is possible that food or foods can be the major cause. Difficulties in controlling their asthma over years in these cases suggested that the causative factors had still not been defined. A "few foods diet", containing only the foods which very rarely cause allergy, was introduced in addition to their usual regimen to be taken for two or three weeks to find out if foods were causing the asthma. Obvious subjective and objective improvement occurred within days, control of the asthma became easier than for years, Peak flow rates increased considerably, and quality of life improved greatly. Improvements were sustained while on the diet and while the causative food or foods were avoided. Blind provocation was not practical, but three exacerbations after re-introduction of specific foods, with continued well-being on avoidance, was accepted as proof. This experience suggests that a hidden allergy or intolerance to a common daily food can cause of chronic asthma, even when there is strong evidence that inhalant allergens are responsible. Enquiry regarding reflux, dyspepsia, or other gut disorders may have been omitted at the first consultation or at follow-up, and patients are most unlikely to associate gut symptoms with their asthma. These cases are presented because food allergy as a hidden cause of chronic asthma is rarely considered, the incidence is unknown, and skin tests and immunology unreliable. A trial of a few foods diet for two or three weeks can be very rewarding, and there is nothing to lose except some weight. A trial in a large group of severe asthmatics could define the true incidence of hidden food allergy in chronic asthma.

